# Excess mortality from cirrhosis by neighbourhood deprivation, aetiology, and clinical presentation: a Swedish register-based cohort study

**DOI:** 10.1016/j.eclinm.2026.103830

**Published:** 2026-03-17

**Authors:** Juan Vaz, Jeffrey V. Lazarus, Hannes Hagström, Ulf Strömberg

**Affiliations:** a School of Public Health and Community Medicine, Institute of Medicine, Sahlgrenska Academy, University of Gothenburg, Sweden; b Department of Medicine, Huddinge, Karolinska Institutet, Stockholm, Sweden; c Department of Medicine, Halland Hospital Halmstad, Halmstad, Sweden; d City University of New York Graduate School of Public Health & Health Policy (CUNY SPH), New York, USA; e Barcelona Institute for Global Health (ISGlobal), Barcelona, Spain; f Unit of Hepatology, Department of Upper GI Diseases, Karolinska University Hospital, Stockholm, Sweden

**Keywords:** Burden of disease, Disparities, Liver disease, Socioeconomic status, Survival

## Abstract

**Background:**

Socioeconomic inequalities are a persistent determinant of health inequity, but their influence on excess mortality from cirrhosis in high-income countries with universal healthcare is not well characterised. We aimed to estimate cirrhosis-related excess mortality across neighbourhood deprivation levels in Sweden.

**Methods:**

We conducted a nationwide, register-based cohort study, including all individuals aged 40 years or older residing in Sweden between 2012 and 2022. The open, underlying cohort was stratified by calendar year, sex, 5-year age groups, and residential neighbourhood. Neighbourhood strata were collapsed into deprivation quintiles, as per the national distribution of inhabitants with a low disposable household income, with Q1 being the least and Q5 the most deprived. Incident cirrhosis cases within the age span 40–74 years were identified using validated diagnostic algorithms, and followed for up to 5 years. Excess mortality rate ratios (EMRRs) were estimated using Poisson regression within a relative survival framework.

**Findings:**

Among 21,583 individuals with incident cirrhosis, 8814 deaths occurred during 65,170 person-years of follow-up. The EMRR for 5-year excess mortality in Q5 versus Q1 was 1.19 (95% CI 1.10–1.29). At the population level, the age-standardised number of excess deaths within 5 years of diagnosis was substantially higher in deprived areas; with 2018 as the year of diagnosis, there were 23 excess deaths per 100,000 inhabitants in Q5 versus 12 in Q1, representing a 95% higher excess mortality burden.

**Interpretation:**

Even within a universal healthcare system, cirrhosis-related excess mortality substantially increases with level of neighbourhood deprivation. Prevention and more optimal post-diagnostic management targeted towards deprived neighbourhoods are urgently needed to reduce avoidable deaths.

**Funding:**

The Swedish Cancer Society (Cancerfonden), The Swedish Research Council for Health, Working life and Welfare (Forte), The Swedish Gastroenterology Fund (Mag-Tarmfonden), The Swedish Research Council (Vetenskapsrådet), The Swedish Society of Medicine, The Swedish Foundation for Transplant and Cancer Research, and Region Stockholm (via a CIMED and clinical researcher award).

**Research in context**

**Evidence before this study**

We searched PubMed and Embase for studies published up to October 2025 using the terms “cirrhosis”, “mortality”, “socioeconomic”, “inequality”, and “deprivation”. Population-based studies from the USA, Denmark, the UK, and Sweden consistently reported social gradients in cirrhosis incidence and mortality, particularly for alcohol-related liver disease (ALD). However, most prior analyses relied on individual-level measures and/or standard survival models, without accounting for background mortality differences across socioeconomic groups. To the best of our knowledge, no nationwide study has estimated cirrhosis-attributable excess mortality using relative survival methods in a high-income country with universal healthcare and extensive welfare safety nets.

**Added value of this study**

Using comprehensive linkage across Swedish national registers, we analysed the records of more than 21,000 individuals aged 40–74 years at cirrhosis diagnosis between 2012 and 2022. Despite comparable cirrhosis severity at diagnosis, individuals in the most deprived neighbourhoods had over twice the incidence and 21% higher excess mortality than those in the least deprived ones. These results demonstrate that neighbourhood deprivation strongly influences avoidable mortality attributable to cirrhosis, reflecting inequities in risk factors, post-diagnostic management, treatment intensity, and health system engagement.

**Implications of all the available evidence**

Socioeconomic deprivation is a powerful and independent determinant of cirrhosis outcomes, even in countries with universal healthcare. The persistence of excess mortality in deprived areas underscores that equal healthcare access does not equate to equitable outcomes. Public health efforts should focus on upstream social determinants of health, including alcohol harm reduction, metabolic risk management, and health literacy, while ensuring equitable access to health surveillance and specialist care. Mapping neighbourhood-level excess mortality can further guide targeted interventions, in line with the European Association for the Study of the Liver–*Lancet* Liver Commission's call for equity-driven liver health strategies across Europe.

## Introduction

Cirrhosis is a leading cause of morbidity and mortality worldwide, with an estimated two million deaths annually.^1^ Although the burden of hepatitis C has declined in many high-income countries due to antiviral therapy use, the incidence of cirrhosis continues to rise, driven by alcohol-related liver disease (ALD) and metabolic dysfunction-associated steatotic liver disease (MASLD).^2^ In Sweden, where healthcare is universally accessible, previous studies have identified socioeconomic disparities in cirrhosis incidence and outcomes.^3^^,^^4^

Socioeconomic status is a key determinant of liver health, influencing the risk of developing cirrhosis and survival post diagnosis.^3^, ^4^, ^5^, ^6^, ^7^ Swedish register data have shown that individuals across socioeconomic strata present with similar rates of cirrhosis with major adverse liver outcomes (MALOs), indicating that differences in survival post diagnosis may not be fully explained by clinical severity at presentation.^4^ This suggests that contextual social determinants of health play an important role in influencing disease trajectory and mortality.^8^^,^^9^

Neighbourhood deprivation, a robust proxy for area-level socioeconomic status, reflects the broader social and environmental conditions in which individuals live.^10^ Studying cirrhosis incidence and mortality across these strata can provide critical insights pertaining to health equity and public policy. Excess mortality, estimated through relative survival methods, can be used to compare observed deaths in individuals living with cirrhosis with expected deaths in the general population, matched by sex, age, calendar year, and deprivation level.^11^, ^12^, ^13^ This approach avoids reliance on potentially misclassified cause-of-death data and accounts for baseline differences in background mortality.^12^^,^^13^

In this nationwide cohort study, we aimed to estimate cirrhosis-related excess mortality across socioeconomic groups in Sweden. The primary focus of the study was to estimate excess mortality associated with cirrhosis across contextual socioeconomic groups, defined at the population level by using neighbourhood deprivation as a proxy. We hypothesised that individuals living in more socioeconomically deprived neighbourhoods would have a higher incidence of cirrhosis and greater excess—and thus potentially avoidable—mortality, with a clear gradient from the least to the most deprived areas.

## Methods

### Study population and data sources

This study included all individuals aged 40 years or older and residing in Sweden between 2012 and 2022 as an open, underlying cohort, and used additional data from the Decoding the Epidemiology of Liver Disease in Sweden (DELIVER) cohort, one of the largest resources worldwide for liver disease epidemiology.^14^ The DELIVER cohort was created through linkage of nationwide Swedish health and demographic registers using the unique personal identity number assigned to all residents. Data sources included the National Patient Register (inpatient and outpatient specialist care, but no primary care visits), the Prescribed Drug Register, the Cancer Register, the Cause of Death Register, and sociodemographic registers from Statistics Sweden. The DELIVER cohort comprises more than 400,000 individuals with liver disease diagnoses recorded between 1964 and 2022.^4^

In this study, we included all incident cases with a first International Classification of Diseases (ICD) code associated with cirrhosis, recorded between 1 January 2012 and 31 December 2022, among individuals aged 40–74 years. The lower age threshold was chosen because cirrhosis in younger individuals in Sweden is more often attributable to autoimmune or rare metabolic liver diseases,^15^ whereas our primary aim was to identify contextual risk patterns relevant to more common liver diseases with preventable or modifiable causes such as ALD, MASLD, and viral hepatitis.^16^ The upper age threshold was set by considering the Organisation for Economic Co-operation and Development/Eurostat definition of avoidable (i.e., preventable and treatable) mortality in high-income countries.^17^ This study focused on excess mortality for up to five years after a cirrhosis diagnosis, meaning that parts of the results were based on mortality in the age-span of 75–79 years. The restriction of follow-up to five years post diagnosis allowed for examination of calendar year trends, with comparable follow-up periods. The study period commencement was set for 2012, as this was the first year for which we have complete mortality data for the entire Swedish population, stratified by the chosen contextual socioeconomic status measure used for this study, which is described below.

We excluded individuals with cirrhosis or decompensation, liver transplantation, or hepatocellular carcinoma that was diagnosed before 2012 (with prevalent cases being identified between 1964 and 2011), individuals with a missing or erroneous personal identity number, and individuals without available neighbourhood-level socioeconomic information required for the classification described below. We also excluded uncertain cases, defined as individuals with ICD codes for cirrhosis-related complications (e.g., ascites or liver failure) but without a registered diagnosis of cirrhosis or chronic liver disease.^4^

### Ethics

This study was approved by the Central Ethical Review Board in Sweden (reference number 2017/1019–31/1). Owing to its register-based design, the requirement for informed consent from the included individuals was waived.

### Procedures

Individuals’ residential area at the time of cirrhosis diagnosis was linked to Statistics Sweden's small-area geographical units, Demografiska StatistikOmråden (DeSO), which launched in 2018 to capture socioeconomic conditions and residential segregation. There are 5984 DeSO units. In 2018, each unit comprised between 600 and 4300 residents, with a median of about 1600. Because the composition of each DeSO is sensitive to demographic change and migration, year-specific DeSO data were applied across the entire study period.

Household income was the main indicator used to construct the DeSO-based deprivation measure applied in this study. Disposable household income was defined as all taxable and tax-exempt income minus taxes and transfers, and standardised for household size and composition using Statistics Sweden's equivalence weights. Low household income was defined as income within the lowest national quartile of the annual distribution of disposable household income. For each calendar year, DeSOs were ranked nationally according to the proportion of residents with a low household income. Based on this ranking, DeSOs were divided into quintiles (Q1–Q5), with Q1 representing the least deprived and Q5 the most deprived areas.^18^ This single deprivation indicator has been shown to have a high explanatory power for mortality and correlates strongly with other proposed indices of multiple deprivation.^19^^,^^20^ The DeSO-based measure thus provides a neighbourhood-level indicator of socioeconomic conditions, which is particularly relevant for identifying geographic inequities and guiding targeted public health interventions.^21^ Each individual's contextual socioeconomic status was determined by their residential DeSO at the time of cirrhosis diagnosis, or, if missing, the year before diagnosis.

Annual population and mortality counts for each DeSO, stratified by calendar year, sex, and 5-year age groups, were obtained from Statistics Sweden. These data were used to construct denominators matched to the case groups by sex, age, year, and DeSO quintiles; this enabled estimation of incidence rates and a direct comparison of observed mortality among individuals living with cirrhosis with the expected mortality in the general population, under equivalent demographic, including contextual socioeconomic, conditions.

For descriptive purposes, we also present selected individual-level sociodemographic characteristics (e.g., region of birth and household income), but these were not used as covariates in the analyses (Appendix page 2). Incidence patterns across these individual-level factors have been previously characterised,^4^ while in this study we focus instead on population groups that are most relevant for future public health initiatives.

ICD codes for cirrhosis diagnoses in the National Patient Register have been previously validated with positive predictive values around 90%.^22^ Because cirrhosis frequently arises from multiple interacting risk factors, we applied a predefined aetiological hierarchy to assign each individual a primary cause of disease and minimise misclassification.^4^ The full list of ICD codes and the aetiological hierarchy is provided in the Appendix (page 3).

MALOs were defined as ascites, variceal bleeding, hepatic encephalopathy, hepatorenal syndrome, liver transplantation, or hepatocellular carcinoma. Events recorded within 90 days of a cirrhosis diagnosis were classified as baseline MALOs. For hepatocellular carcinoma, the window was extended to 180 days to account for small tumours potentially being present at diagnosis but detected during the initial evaluation period.^4^ Cirrhosis severity at diagnosis was operationalised using the presence or absence of MALOs as proxy markers of decompensation. Individuals without recorded complications were classified as having compensated cirrhosis without hepatocellular carcinoma, whereas those with evidence of complications were considered to have decompensated disease and/or hepatocellular carcinoma.

Data on comorbidities were retrieved from the National Patient Register and, where relevant, from the Prescribed Drug Register (Appendix page 3). Lifestyle-related variables such as alcohol use, body mass index, or tobacco smoking are not recorded in the nationwide registers and were therefore not available.

### Outcomes

The main outcome was excess mortality associated with cirrhosis across socioeconomic strata. Exploratory outcomes included the incidence of all-cause cirrhosis, cirrhosis with and without a MALO, and cirrhosis by aetiology across neighbourhood deprivation levels.

### Statistics

Continuous variables were summarised as medians with IQRs and categorical variables as counts and percentages.

Excess mortality was defined as the number of deaths observed among individuals diagnosed with cirrhosis beyond those expected in the general population with the same age, sex, calendar year, and neighbourhood deprivation profile. Individuals were followed from the date of diagnosis until whichever date occurred first among death, emigration, 5 years after diagnosis, or 31 December 2023. Emigration was treated as censoring. Loss to follow-up was minimal due to complete national register coverage.

Excess mortality was modelled using relative survival methods, with population life tables being matched by age, sex, calendar year, and quintile.^11^ Excess mortality rates and excess mortality rate ratios (EMRRs) at 1 year and 5 years after cirrhosis diagnosis were estimated using Poisson regression models. EMRRs compare cirrhosis-attributable mortality between deprivation quintiles, quantifying differences beyond those expected from underlying socioeconomic gradients in background population mortality.

Analyses estimating 1-year EMRRs included the entire cohort (2012–2022), whereas 5-year analyses were restricted to individuals diagnosed between 2012 and 2018 to ensure complete follow-up. We focused on excess mortality within 1 year and 5 years of cirrhosis diagnosis, because these periods capture the highest excess mortality burden, particularly during the first year after diagnosis, when preventable deaths are most frequent. While long-term survival of individuals with cirrhosis has improved over time,^1^^,^^2^ especially following advances in antiviral therapy, longer follow-up would increasingly reflect competing causes of death and dilute cirrhosis-attributable excess mortality. Both models were adjusted for age group, sex, calendar period (2012–2015, 2016–2019, 2020–2022 for 1-year analyses; 2012–2015 and 2016–2018 for 5-year analyses), and cirrhosis aetiology due to their established prognostic relevance and potential confounding of socioeconomic associations. 5-year models additionally included the number of follow-up years as a covariate.

Baseline MALOs were included as a proxy for clinical severity at diagnosis. Because MALOs may partly reflect delayed diagnosis and thus lie on the causal pathway between deprivation and mortality, we additionally performed sensitivity analyses excluding MALOs (Appendix pages 6–7). To assess potential effect modification, interaction terms between quintile and cirrhosis aetiology were included in the relative survival models. A global test of interaction was performed using Wald tests. Model fit was evaluated by assessing overdispersion; as no substantial overdispersion was detected, Poisson models with robust standard errors were used throughout.

Age-standardised incidence rate (ASIR) of cirrhosis among individuals aged 40–70 years, with 95% CIs, were estimated per 100,000 persons using direct standardisation to the 2013 Revised European Standard Population.^23^ ASIRs were estimated by quintile. Associations between socioeconomic deprivation and cirrhosis incidence were analysed using Poisson regression adjusted for sex, age group, and calendar year, all entered as categorical variables.

We also estimated the number of excess deaths attributable to cirrhosis per 100,000 inhabitants during the five years following a cirrhosis diagnosis, stratified by calendar year (i.e., 2012, 2015, and 2018), age group, and quintile. Detailed statistical methods, including life-table expansion, age-standardisation procedures, and derivation of excess deaths, are provided in the Appendix (page 2).

Missing data were very limited for most variables. Individuals without available DeSO data (n = 156) were excluded from the analyses, primarily because deprivation could not be assigned for individuals with protected residential addresses. Given the small proportion of excluded individuals, neither multiple imputation nor inclusion of a separate missing category was considered appropriate as the potential impact of the missing data on the results was expected to be minimal.

All relative survival and excess mortality analyses were performed using the *strs* command in Stata (version 19; StataCorp, College Station, Texas, USA).^11^

### Role of funding source

The funders had no role in study design, data collection, data analysis, data interpretation, or writing of the report.

## Results

**Fig. 1:**
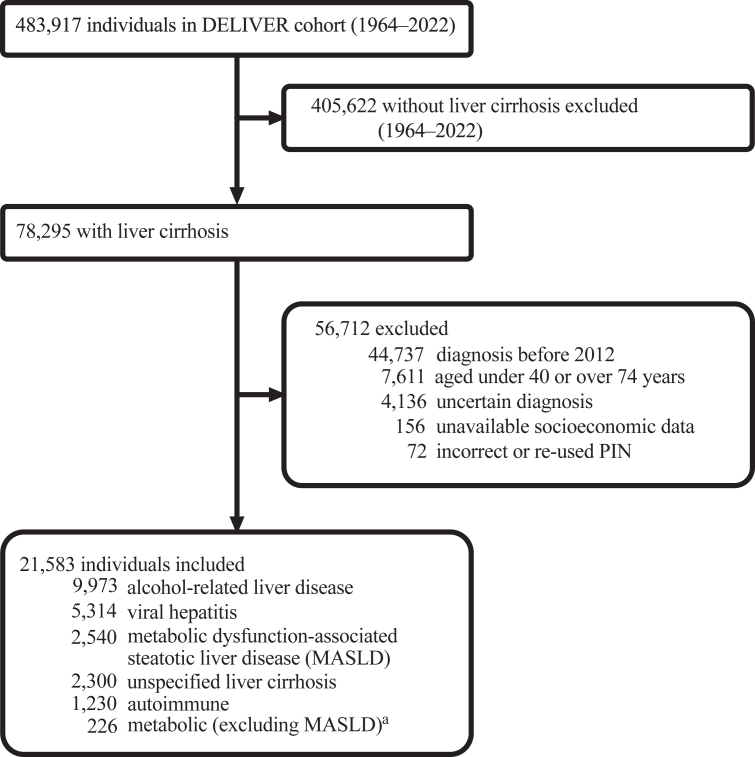
Individuals aged 40–74 years with a first diagnosis of cirrhosis registered in the Decoding the Epidemiology of Liver Disease in Sweden (DELIVER) cohort, 2012–2022. Individuals with incident cirrhosis were classified into one of six aetiological categories. MASLD = metabolic dysfunction-associated steatotic liver disease. PIN = personal identity number. ^a^ Including α1-antitrypsin deficiency, haemochromatosis, and Wilson's disease but not MASLD.

After exclusions, 21,583 individuals diagnosed with cirrhosis were identified (Fig. 1); the median age at diagnosis was 63 years (IQR 56–69) and 7617 (35%) were female (Table 1). Most individuals (n = 18,786 [87%]) were born in the Nordic region. 2948 (14%) individuals were in Q1, and 5722 (26%) were in Q5, indicating a clear socioeconomic gradient.

**Table 1:** Baseline characteristics of 21,583 individuals aged 40–74 years diagnosed with cirrhosis in Sweden between 2012 and 2022.

	Neighbourhood deprivation level (quintiles)					Total
	Q1 (least)	Q2	Q3	Q4	Q5 (most)	
	2948 (14)	3857 (18)	4271 (20)	4785 (22)	5722 (27)	21,583 (100)
**Sex**						
Male	1859 (63)	2478 (64)	2804 (66)	3133 (65)	3692 (65)	13,966 (65)
Female	1089 (37)	1379 (36)	1467 (34)	1652 (35)	2030 (35)	7617 (35)
**Median age**	64 (57–69)	63 (56–69)	63 (56–69)	63 (56–69)	62 (55–68)	63 (56–69)
**Age group**						
40–44	92 (3)	131 (3)	118 (3)	178 (4)	255 (5)	774 (4)
45–49	165 (6)	236 (6)	245 (6)	311 (7)	434 (8)	1391 (6)
50–54	312 (11)	410 (11)	467 (11)	526 (11)	711 (12)	2426 (11)
55–59	426 (14)	588 (15)	691 (16)	758 (16)	965 (17)	3428 (16)
60–64	587 (20)	754 (20)	820 (19)	990 (20)	1111 (19)	4262 (20)
69–70	703 (24)	849 (22)	1048 (24)	1015 (21)	1248 (22)	4863 (22)
70–74	663 (22)	889 (23)	882 (21)	1007 (21)	998 (17)	4439 (21)
**Region of birth**						
Nordic	2705 (92)	3543 (92)	3892 (91)	4243 (89)	4403 (77)	18,786 (87)
Non-Nordic	243 (8)	314 (8)	379 (9)	542 (11)	1319 (23)	2797 (13)
**Educational level**						
High (>12 years)	902 (30)	916 (24)	862 (20)	787 (16)	787 (14)	4254 (20)
Medium (10–12 years)	1384 (47)	1928 (50)	2162 (51)	2482 (52)	2823 (49)	10,779 (50)
Low (≤9 years)	637 (22)	976 (25)	1193 (28)	1426 (30)	1911 (33)	6143 (28)
Unknown	25 (1)	37 (1)	54 (1)	90 (2)	201 (4)	407 (2)
**Household income**						
High	1102 (37)	1003 (26)	804 (19)	691 (14)	464 (8)	4064 (19)
Medium	1359 (46)	1863 (48)	2022 (47)	2171 (46)	2190 (38)	9605 (44)
Low	487 (17)	991 (26)	1445 (34)	1923 (40)	3068 (54)	7914 (37)
**Aetiology**						
Viral hepatitis	445 (15)	783 (20)	941 (22)	1205 (25)	1940 (34)	5314 (24)
ALD	1550 (53)	1908 (50)	2034 (47)	2211 (46)	2270 (40)	9973 (46)
MASLD	315 (11)	436 (11)	533 (12)	586 (12)	670 (12)	2540 (12)
Autoimmune	215 (7)	242 (6)	247 (6)	273 (6)	253 (4)	1230 (6)
Metabolic^a^	42 (1)	36 (1)	46 (1)	55 (1)	47 (1)	226 (1)
Unspecified	381 (13)	452 (12)	533 (12)	455 (10)	542 (9)	2300 (11)
**MALO at diagnosis**	1395 (47)	1747 (45)	1947 (46)	2086 (44)	2373 (41)	9548 (44)
HCC	162 (5)	230 (6)	284 (7)	338 (7)	398 (7)	1412 (7)
**Comorbidities**						
Arterial hypertension	1052 (36)	1407 (36)	1533 (36)	1669 (35)	1906 (33)	7567 (35)
Type 2 diabetes	839 (28)	1164 (30)	1299 (30)	1474 (31)	1825 (32)	6601 (31)
Obesity	316 (11)	453 (12)	520 (12)	608 (13)	762 (13)	2659 (12)
Hyperlipidaemia	944 (32)	1243 (32)	1338 (31)	1531 (32)	1735 (30)	6791 (31)
CAD	262 (9)	362 (9)	433 (10)	517 (11)	630 (11)	2204 (10)
CVD	146 (5)	202 (5)	229 (5)	300 (6)	364 (6)	1241 (6)
CKD	169 (6)	232 (6)	245 (6)	299 (6)	371 (6)	1316 (6)
COPD	225 (8)	327 (8)	415 (10)	439 (9)	614 (11)	2020 (9)
Depression or anxiety	224 (8)	364 (9)	369 (9)	463 (10)	537 (9)	1957 (9)
Non-HCC cancer	279 (9)	355 (9)	392 (9)	393 (8)	377 (7)	1796 (8)

ALD = alcohol-related liver disease. CAD = coronary artery disease. CKD = chronic kidney disease. CVD = cerebrovascular disease. COPD = chronic obstructive pulmonary disease. HCC = hepatocellular carcinoma. MALO = major adverse liver outcome. MASLD = metabolic dysfunction-associated steatotic liver disease.

a Including alpha-1-antitrypsin deficiency, haemochromatosis, and Wilson's disease but not MASLD.

ALD was the most common underlying cause of cirrhosis (n = 9973 [46%]), followed by viral hepatitis (n = 5314 [24%]), and MASLD (n = 2540 [12%]). Viral hepatitis-related cirrhosis was most frequently occurring among individuals residing Q5, whereas the opposite pattern was observed for ALD. At the time of cirrhosis diagnosis, 9548 (44%) individuals presented with at least one MALO, including 1412 (7%) with hepatocellular carcinoma. Individual-level characteristics stratified by sex are presented in the Appendix (page 4).

**Fig. 2:**
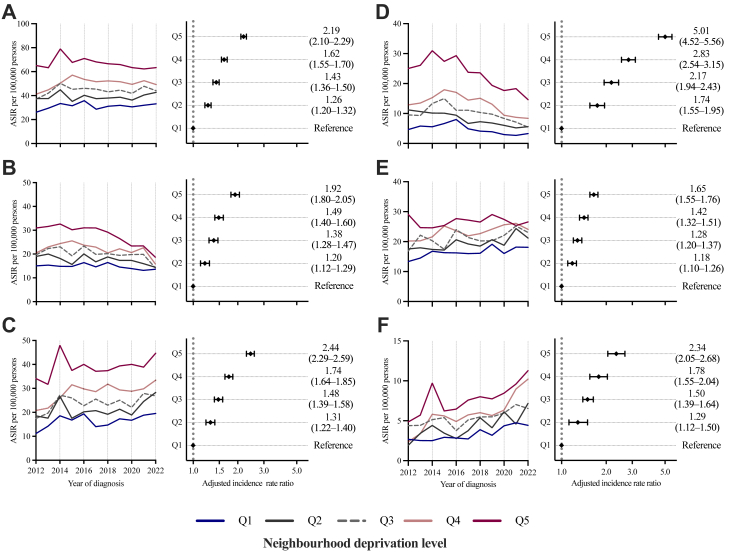
ASIRs and incidence rate ratios adjusted for sex, age, and calendar year for: all-cause cirrhosis (A); cirrhosis with and without MALOs (B and C); viral hepatitis-related cirrhosis (D); ALD-related cirrhosis (E); and MASLD-related cirrhosis (F), among individuals aged 40–74 years in Sweden, 2012–2022. Estimates are stratified by neighbourhood deprivation level, from least (Q1) to most deprived (Q5). Incidence rates were age-standardised to the 2013 Revised European Standard Population. Note that the scale of the y-axes varies between panels. ALD = alcohol-related liver disease. ASIR = age-standardised incidence rate. IRR = incidence rate ratio. MALO = major adverse liver outcome. MASLD = metabolic dysfunction-associated steatotic liver disease.

The ASIR of cirrhosis increased from 40.8 per 100,000 inhabitants (95% CI 38.9–42.8) in 2012 to 45.3 (43.3–47.3) in 2022. Individuals in Q5 had an ASIR of 67.6 (CI 65.8–69.3) versus 31.1 (30.0–32.3) in Q1, corresponding to an incidence rate ratio of 2.19 (2.10–2.29; Fig. 2). This socioeconomic gradient was consistent across all major aetiologies of cirrhosis (ALD, viral hepatitis, and MASLD), with higher ASIRs and incidence rate ratios being observed in the most deprived quintiles.

**Fig. 3:**
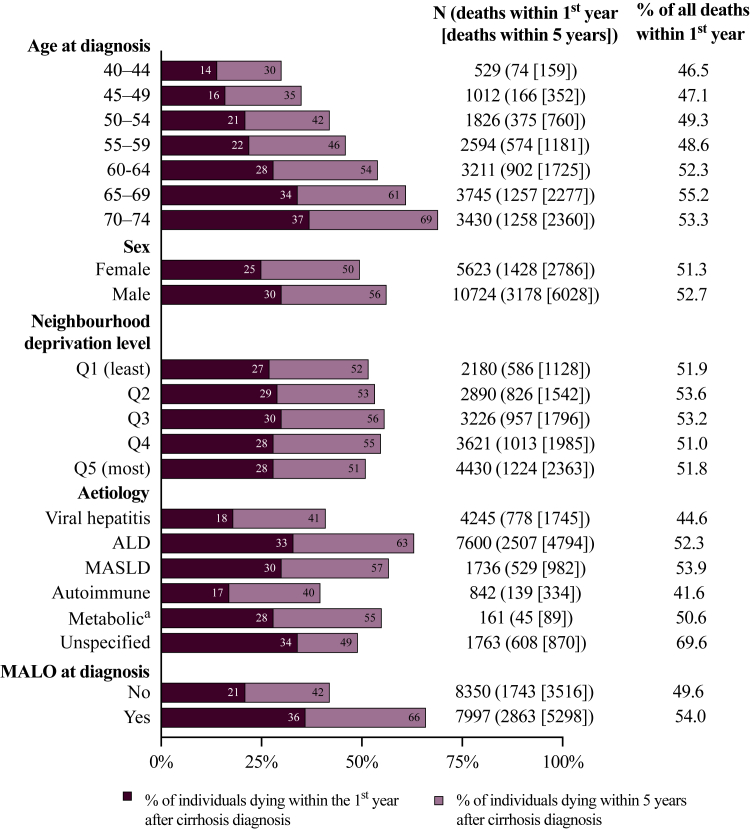
One- and five-year mortality after cirrhosis diagnosis among individuals aged 40–74 years in Sweden, 2012–2018. Proportions of individuals dying within the first year (dark bars) and within five years (light bars) post diagnosis are shown, stratified by age at diagnosis, sex, neighbourhood deprivation level, aetiology, and occurrence of a MALO at diagnosis. Numbers within the bars represented the proportion of the total group for each category. Numbers to the right of each bar indicate the total number of individuals, the number of deaths within one year (in parentheses), and the number of deaths within five years (in brackets). The percentage of all deaths that occurred during the first follow-up year is also shown. ALD = alcohol-related liver disease. MALO = major adverse liver outcome. MASLD = metabolic dysfunction-associated steatotic liver disease. ^a^ Including α1-antitrypsin deficiency, haemochromatosis, and Wilson's disease but not MASLD.

A total of 16,347 individuals were diagnosed with cirrhosis between 2012 and 2018, among whom 8814 deaths (54%) were registered within 5 years of cirrhosis diagnosis, corresponding to 65,170 person-years of observation and a median follow-up of 3.2 years. Of these deaths, 4606—corresponding to 52% of all deaths that occurred within 5 years of diagnosis—took place during the first year after diagnosis. The number and proportion of deaths that occurred within the first and fifth year of follow-up among these 13,347 individuals are shown in Fig. 3; although the crude proportions of these deaths appeared similar across quintiles, this pattern likely reflects confounding. As shown in Table 1, individuals in Q5 were younger but had a higher prevalence of viral hepatitis-related cirrhosis and a slightly lower prevalence of MALOs.

**Fig. 4:**
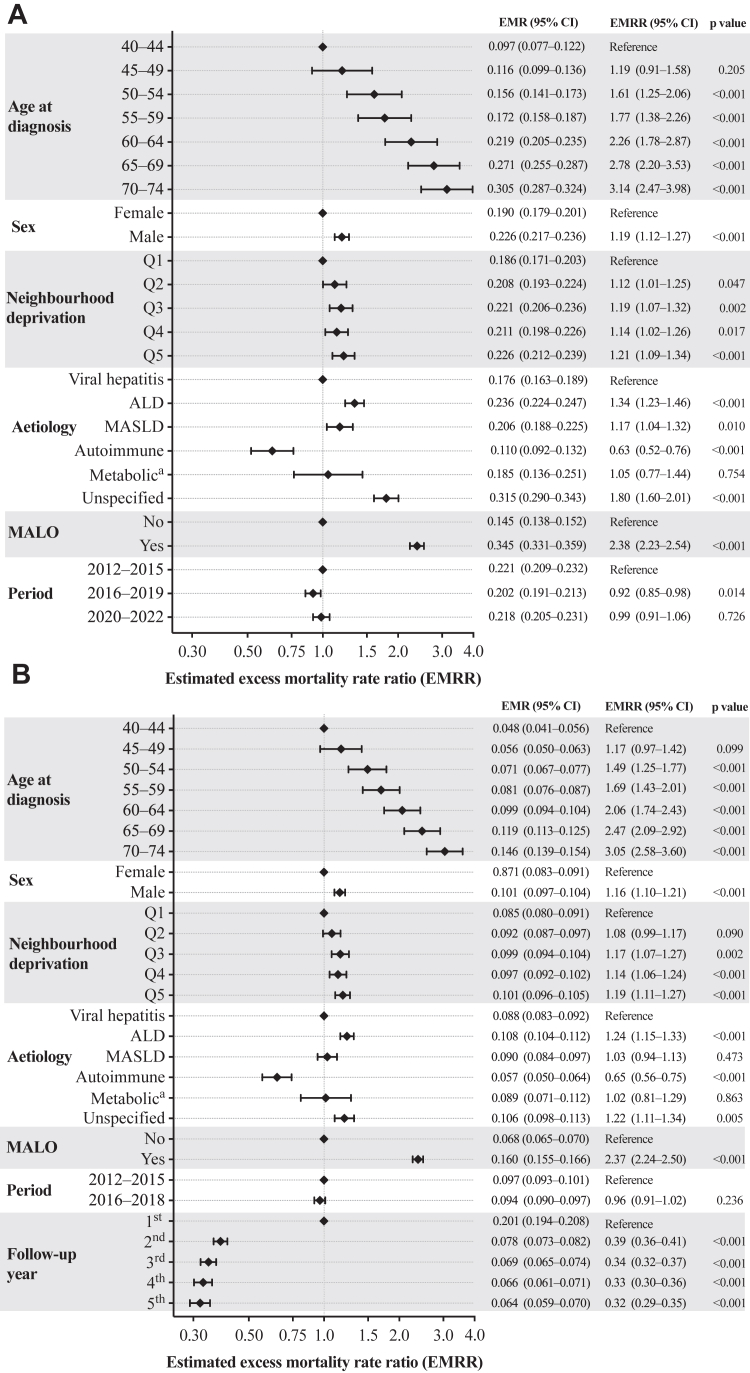
Estimated excess mortality rate (EMR) and excess mortality rate ratio (EMRR) among individuals aged 40–74 years diagnosed with cirrhosis in Sweden, 2012–2022. Panel A shows EMR and EMRR estimates during the first year post cirrhosis diagnosis for the entire study period (2012–2022). Panel B presents estimates for individuals diagnosed between 2012 and 2018 with complete five-year follow-up data. Estimates are derived from multivariable Poisson regression models adjusted for all variables shown. ALD = alcohol-related liver disease. MALO = major adverse liver outcome. MASLD = metabolic dysfunction-associated steatotic liver disease. ^a^ Including α1-antitrypsin deficiency, haemochromatosis, and Wilson's disease but not MASLD.

In the fully adjusted models, excess mortality 1 year and 5 years after diagnosis was significantly associated with neighbourhood deprivation, even after accounting for age, sex, aetiology, and cirrhosis severity at presentation (Fig. 4). Compared with individuals in Q1, those in Q5 had an EMRR of 1.21 (95% CI 1.11–1.31) within the first year and 1.19 (1.11–1.27) at 5 years of follow-up. Compared to individuals with viral hepatitis-associated cirrhosis, those with ALD- and MASLD-associated, or unspecified, cirrhosis had higher EMMRs within 1 year of follow-up, with this also being the case for those with ALD-associated and unspecified cirrhosis at 5 years of follow-up.

Excess mortality increased with age; for individuals aged 70–74 years versus 40–44 years the EMRR was 3.05 (95% CI 2.58–3.60) at 5 years of follow-up. Cirrhosis severity at diagnosis was a key prognostic determinant; for individuals with a baseline MALO verus those without one the EMRR was 2.37 (2.24–2.50) at 5 years of follow-up.

There was no statistically significant interaction between neighbourhood deprivation and cirrhosis aetiology (global test p = 0.26), suggesting that the observed socioeconomic gradient in excess mortality was broadly consistent across major aetiological groups.

Temporal trends showed a modest reduction in the 1-year of follow-up excess mortality from 2012 to 2015 and 2016–2018; however, this improvement did not persist into the later period of 2020–2022. By contrast, the 5-year of follow-up model did not demonstrate any statistically significant improvement between 2012–2015 and 2016–2018. Consistent with the observed mortality data from 2012 to 2018 (Fig. 3), EMRRs decreased progressively with increased follow-up time.

**Fig. 5:**
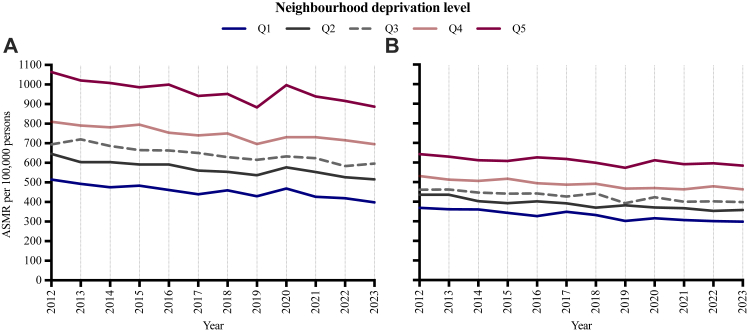
Age-standardised mortality rates (ASMR) among men (A) and women (B) aged 40–79 years in Sweden, 2012–2023. Estimates are stratified by neighbourhood deprivation level, from least (Q1) to most deprived (Q5). Mortality rates were age-standardised to the 2013 Revised European Standard Population.

**Fig. 6:**
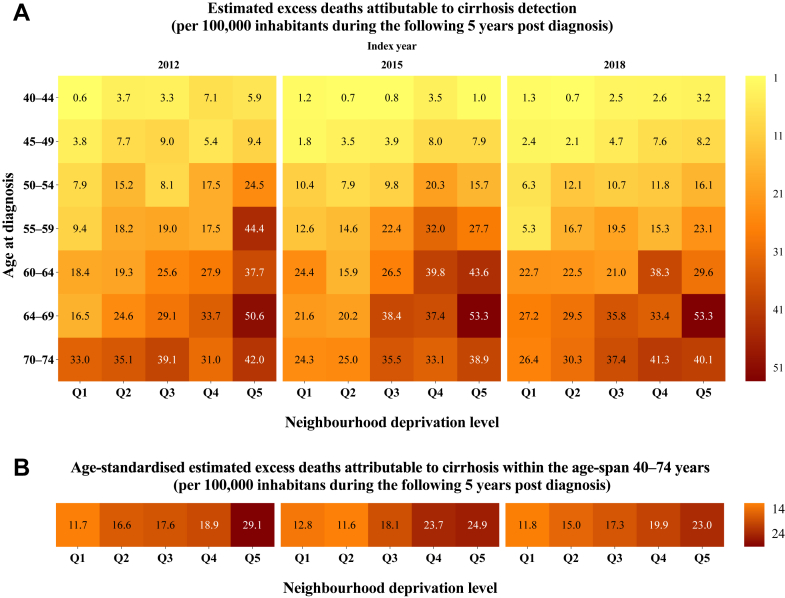
Age-specific (A) and age-standardised (B) estimates of excess deaths attributable to cirrhosis among individuals aged 40–74 years in Sweden with index year 2012, 2015, and 2018, stratified by neighbourhood deprivation level, from least (Q1) to most deprived (Q5). The figure presents the expected number of excess deaths per 100,000 inhabitants within five years following a cirrhosis diagnosis. For example, with 2018 as the index year, the estimates represent a 95% higher excess mortality burden in Q5 versus Q1 (23/11.8 = 1.95) within five-years among individuals with cirrhosis aged 40–74 years. For the Swedish population, the corresponding estimated number of excess deaths became 177 (769,890 inhabitants in Q5) and 108 (915,839 inhabitants in Q1). Estimates were age-standardised to the 2013 Revised European Standard Population.

For reference, the overall age-standardised mortality rate among individuals aged 40–79 years in Sweden between 2012 and 2023 is presented in Fig. 5. At the population level, the estimated number of excess deaths attributable to cirrhosis within 5 years of diagnosis was substantially higher in socioeconomically deprived areas. With 2018 as the year of diagnosis, there were 23 excess deaths per 100,000 inhabitants in Q5 versus 11.8 excess deaths per 100,000 inhabitants in Q1, representing a 95% higher excess mortality burden (Fig. 6). This socioeconomic gradient was evident across 2012, 2015, and 2018 as index years, although the age-standardised number of expected excess deaths declined in Q4 and Q5 for individuals diagnosed in 2015 and 2018, relative to those diagnosed in 2012. Specifically, the number of excess deaths in Q5 decreased markedly among individuals aged 50–64 years at diagnosis in 2018, compared with 2012, whereas such rates remained largely stable among those aged 65 years or older.

## Discussion

This large, register-based cohort study provides comprehensive evidence of socioeconomic disparities in cirrhosis-related excess mortality in Sweden. Despite universal healthcare access,^24^ individuals residing in Q5 have more than twice the incidence of cirrhosis and 21% and 19% higher excess mortality at 1 year and 5 years after diagnosis, respectively, compared with those residing in Q1. These inequalities, which affected the number of excess deaths, were not explained by differences in age at diagnosis, sex, cirrhosis aetiology, or the occurrence of MALOs at presentation, underscoring the impact of contextual socioeconomic factors on disease trajectory, beyond individual-level risk.

The distribution of cirrhosis aetiologies revealed marked socioeconomic differences, with the steepest incidence gradient being observed for viral hepatitis. The incidence of viral hepatitis-related cirrhosis declined substantially in Q4 and Q5 following the introduction of universal access to highly effective hepatitis C treatment in 2016, regardless of liver fibrosis stage.^25^ This pattern mirrors previous findings in Sweden among individuals living with a low income, indicating that equitable access to antiviral therapy has helped to narrow socioeconomic gaps in hepatitis C-related liver disease outcomes.^4^

By contrast, the incidence of MASLD-related cirrhosis increased across all socioeconomic strata, with the most pronounced rise being seen among individuals living in Q4 and Q5. The incidence of cirrhosis without MALOs also increased across all socioeconomic groups, particularly in Q4 and Q5, which may reflect earlier disease detection due to widespread hepatitis C treatment or an increasing incidence of MASLD with compensated disease at diagnosis. Although the incidence of cirrhosis with MALOs decreased substantially among Q4 and Q5, these populations continued to experience the highest excess mortality post diagnosis. This pattern indicates that factors beyond cirrhosis severity at presentation drive the observed survival disparities. Even after adjusting for MALO occurrence at baseline, age, sex, and aetiology, excess mortality remained significantly elevated in Q3, Q4, and Q5.

Because baseline MALOs may partly capture delayed diagnosis and thus represent potential mediators rather than pure confounders, adjustment for MALOs may be conservative. However, socioeconomic gradients in excess mortality persisted in sensitivity analyses excluding MALOs, suggesting that inequalities are not fully explained by differences in disease severity at presentation.

Although later-stage diagnosis in more deprived areas could plausibly contribute to higher excess mortality, we accounted for clinical severity at presentation using MALOs as a proxy for decompensated disease. The persistence of socioeconomic gradients after adjustment for MALOs suggests that differences in stage at diagnosis do not fully explain the observed disparities. Nevertheless, we cannot exclude the possibility that more subtle differences in disease severity or unmeasured markers of frailty at diagnosis may contribute to the excess mortality burden in deprived populations.

These findings suggest that differences in post-diagnostic management, access to follow-up care, and treatment intensity may contribute to the excess mortality burden in these populations. Potential explanations include delayed recognition of disease progression, variations in referral pathways to specialist care, differences in access to liver transplantation, or disparities in the management of complications and comorbidities.^8^^,^^9^^,^^26^ Lower health literacy, barriers in communication, stigma, or reduced trust in healthcare systems may further hinder effective engagement with care and adherence to treatment recommendations.^16^ Additionally, inadequate control of precipitating factors, such as ongoing alcohol overconsumption or poorly managed metabolic risk components, could exacerbate disease progression and worsen survival outcomes.^27^ The persistence of socioeconomic gradients in excess mortality despite similar clinical severity at diagnosis underscores the need for targeted interventions to improve factors like health literacy in high-incidence populations, and optimise long-term management after a cirrhosis diagnosis.

Although our study demonstrates clear socioeconomic gradients in excess mortality after cirrhosis diagnosis, it is important to emphasise that we did not directly measure healthcare utilisation, such as specialist follow-up, surveillance adherence, referral pathways, or treatment intensity. Our findings, therefore, reflect differences in outcomes rather than observed differences in care delivery.

Nevertheless, the persistence of excess mortality despite similar clinical severity at diagnosis suggests that standardised healthcare delivery within a universal, tax-funded system may not fully capture the needs of socially vulnerable populations. Within National Health Service (NHS)-based systems, equal entitlement to care does not necessarily translate into equitable outcomes. Differences in post-diagnostic trajectories may plausibly arise from challenges in navigating complex care pathways, barriers to sustained engagement with long-term management, or mismatches between uniform care models and heterogeneous social needs. These mechanisms should be interpreted as hypotheses rather than directly observed processes in the present study.

Notably, the neighbourhood deprivation measure applied here is comparable to small-area deprivation indices used in the UK,^19^ such as the Index of Multiple Deprivation (https://assets.publishing.service.gov.uk/media/5d8e26f6ed915d5570c6cc55/IoD2019_Statistical_Release.pdf), strengthening the relevance of our findings for other NHS-based healthcare systems.

Future research should focus on identifying the key points along the disease trajectory where inequalities emerge, such as surveillance, treatment initiation, or palliative care, and how these can be mitigated through system-level improvements. Understanding these pathways will be essential for developing equitable strategies to reduce preventable mortality from cirrhosis in socioeconomically disadvantaged populations.

Our findings reinforce a consistent pattern observed internationally: socioeconomic deprivation remains a powerful determinant of survival after cirrhosis diagnosis, even in high-income settings with universal healthcare coverage. Studies from the UK have shown that individuals living in Q5 areas not only had a higher incidence of ALD but also a 16–22% higher mortality risk compared with those in Q1.^6^^,^^28^ Similarly, Danish national registry data demonstrated that socioeconomic disadvantage was associated with a higher cirrhosis incidence and poorer long-term survival.^7^^,^^29^ In general, inequities in alcohol-related mortality, including that of ALD-related cirrhosis have been reported in several European countries.^30^ Additionally, in the USA, where insurance coverage mediates healthcare access, a lower income has been linked to a two-fold higher liver-related mortality risk among individuals with chronic liver disease.^31^

The persistence of these inequalities across diverse healthcare systems indicates that universal access alone cannot eliminate survival gaps. Such disparities may be perpetuated by other factors like a delayed diagnosis, fragmented care coordination, and psychosocial barriers that limit sustained healthcare engagement.^8^^,^^9^^,^^27^ Sweden's comprehensive, tax-funded healthcare system and ample safety nets illustrate that even equitable formal healthcare access cannot fully compensate for contextual socioeconomic disadvantages.^24^

Multilevel interventions are therefore needed. At the clinical level, earlier detection of at-risk individuals through non-invasive fibrosis screening in primary care, particularly among people living with metabolic- or alcohol-related risk factors, could enable a timely referral and prevent decompensation.^32^ Structured follow-up programmes, including nurse-led coordination, telehealth support, and active recall strategies, should be prioritised for individuals in socioeconomically deprived areas.^33^ At the community level, initiatives aimed at improving health literacy, reducing stigma, and strengthening trust in healthcare services are essential to foster care adherence and early diagnoses.^16^ Co-designed educational campaigns with patient organisations and community leaders can help to address cultural and social barriers.

At the policy level, upstream determinants of health must be addressed.^34^ Alcohol control measures such as taxation, minimum unit pricing, and advertising restrictions have demonstrably reduced liver-related hospitalisations and deaths in Scotland.^35^ Similarly, public health strategies to combat unhealthy diets and physical inactivity, to enable improved weight management, are critical to curb the rising MASLD burden, which disproportionately affects under-resourced populations.^36^ Our results also indicate that universal hepatitis C treatment can decrease social gradients in liver disease outcomes.

From a public health standpoint, mapping excess deaths across neighbourhoods can identify hot spots where intensified prevention and improved post-diagnostic care could have the greatest impact. Integrating such spatial insights into national liver health strategies would align with the European Association for the Study of the Liver–*Lancet* Liver Commission's framework, which calls for a coordinated European response to liver disease that places the social determinants of health and equity at its centre.^16^

These results highlight that reducing preventable deaths from cirrhosis requires not only medical innovation but also social and policy action to ensure that the benefits of early diagnosis and effective treatment reach all population groups.

Key strengths of this study include its nationwide, population-based design and comprehensive linkage across high-quality Swedish registers, and the use of relative survival models to estimate cirrhosis-attributable excess mortality while accounting for background mortality differences. The large sample size allowed for precise estimation of incidence and EMRRs.

A major methodological strength is the application of excess mortality analysis, which provides a more comprehensive measure of disease impact than conventional survival models. Unlike cause-specific survival analyses that rely on potentially inaccurate death certification, relative survival compares observed survival among individuals living with cirrhosis to expected survival in the general population, thereby capturing the total mortality burden attributable to the disease. This approach also adjusts for baseline mortality differences linked to socioeconomic status, isolating mortality beyond that expected from socioeconomic disadvantage alone. Consequently, it allows for the identification of residual inequalities that are likely driven by healthcare access, disease management, or behavioural and environmental factors.

Several limitations should be acknowledged. Although individual-level socioeconomic variables were available in Swedish registers, we deliberately focused on neighbourhood-level deprivation to identify population segments and geographical areas where targeted interventions may have the greatest public health impact. This contextual approach aligns with international practice and supports planning of area-based strategies rather than individual risk stratification alone. Nevertheless, neighbourhood deprivation does not fully capture individual socioeconomic position and may introduce ecological bias if interpreted at the individual level. Conversely, focusing solely on individual characteristics risks the individualistic fallacy, whereby contextual influences on health are overlooked. Our findings should, therefore, be interpreted as reflecting area-level socioeconomic gradients in cirrhosis-related excess mortality rather than individual-level causal effects.

Moreover, reliance on ICD coding may have introduced misclassification of cirrhosis aetiologies, particularly MASLD, which remains under-recognised in administrative data despite validated algorithms. In approximately 11% of cases, no clear underlying cause of cirrhosis could be assigned, although data linkage procedures reduced this proportion when compared with previous registry studies.^3^^,^^15^ Additionally, the absence of primary care data may have led to an under ascertainment of early or compensated cirrhosis. Finally, a relative survival analysis assumes that population life tables accurately represent expected mortality within each socioeconomic stratum; deviations from this assumption or differences in comorbidity burden may thus result in minor bias.

Despite universal healthcare, this nationwide study shows that individuals living in Sweden's most socioeconomically deprived neighbourhoods experience the highest incidence of cirrhosis and the greatest excess mortality pertaining to this diagnosis. These findings indicate that socioeconomic context is closely linked to the risk of developing cirrhosis and dying from its complications, although the underlying mechanisms cannot be fully disentangled with the presented data. A substantial proportion of cirrhosis-related deaths each year are therefore likely preventable, representing hundreds of avoidable deaths nationwide.

## Contributors

The work reported in the article has been performed by the authors, unless clearly specified in the text. Specific author contributions: JV: Conceptualization, Methodology, Formal analysis, Investigation, Visualization, Data acquisition, Writing–Original Draft. JVL: Writing–review and editing. HH: Conceptualization, Methodology, Formal analysis, Investigation, Data acquisition, Funding acquisition, Writing–Original Draft, Main Supervision. US: Conceptualization, Methodology, Formal analysis, Data acquisition, Writing–Original Draft, Funding acquisition, Main Supervision. HH and US contributed equally to the Main Supervision. JV and HH had full access to all of the data in the study and take responsibility for its integrity. Data verification was performed independently by both authors. All authors contributed to, read, and approved of the final version of the manuscript.

## Data sharing statement

Datasets generated and analysed during the current study are not publicly available due to legal restrictions, but additional analyses may be requested from the corresponding author upon reasonable request. External researchers may request the raw data from Swedish healthcare registers and perform additional analyses.

## Declaration of interests

JV has received consulting fees from Roche and Astra Zeneca and a research grant from Eisai, outside of this work. JV has also received grants for this study from The Swedish Society of Medicine, The Swedish Foundation for Transplant and Cancer Research, and The Swedish Gastroenterology Fund. JVL has received grants to his institutions from Gilead Sciences, Pfizer, Echosens, Boehringer Ingelheim, Madrigal Pharmaceuticals, MSD, and Novo Nordisk, consulting fees from GSK, Echosens, Novo Nordisk, and Takeda, and honoraria for lectures from Echosens, Gilead Sciences, GSK, MSD, Novo Nordisk, and Pfizer, outside of this work. HH's institutions have received research funding from Astra Zeneca, Echosens, Gilead, Intercept, MSD, Novo Nordisk, Takeda, and Pfizer. He has served as consultant, speaker or on advisory boards for Astra Zeneca, Boehringer Ingelheim, Bristol Myers-Squibb, GSK, Echosens, Ipsen, MSD, and Novo Nordisk and has been part of hepatic events adjudication committees for Arrowhead, Boehringer Ingelheim, KOWA and GW Pharma. This has all been outside of this work. US has received grants to his institution from The Swedish Cancer Society and The Swedish Research Council for Health, Working life and Welfare, which partially financed this study.
