# Supplementary material for: Excess mortality from cirrhosis by neighbourhood deprivation, aetiology, and clinical presentation: a Swedish register-based cohort study
Source: eClinicalMedicine. 2026 Mar 17;94:103830. doi: 10.1016/j.eclinm.2026.103830 (PMC13011078; doi:10.1016/j.eclinm.2026.103830)
Supplement: Supplementary Tables [file mmc1.docx]

**Excess mortality from cirrhosis by neighbourhood deprivation, aetiology, and clinical presentation: a Swedish register-based cohort study**

Juan Vaz, Jeffrey V Lazarus, Hannes Hagström, Ulf Strömberg

Supplementary Appendix

Table of contents

[Detailed Statistical Methods 2](#_Toc222127607)

[Table S1. Sociodemographic characteristics 3](#_Toc222127608)

[Table S2. Clinical characteristics 4](#_Toc222127609)

[Table S3. Baseline characteristics of 21,583 individuals aged 40–74 years diagnosed with cirrhosis in Sweden between 2012–2022, stratified by sex 5](#_Toc222127610)

[Table S4. Adjusted estimated excess mortality rate ratio (EMRR) at one and five years after cirrhosis diagnosis among individuals aged 40–74 years in Sweden, 2012–2022 6](#_Toc222127611)

# **Detailed Statistical Methods**

Population life tables and linkage

Population and mortality count data from Statistics Sweden were originally available in five-year age bands. To achieve finer alignment between the cirrhosis cohort and the general population, these data were expanded to single-year age bands up to age 79 (i.e., the maximum age attainable by individuals diagnosed with cirrhosis at age 74 who had a five-year follow-up). The same mortality rate and survival probability were assigned to all ages within each five-year group.

The expanded population life tables were then linked to the cirrhosis cohort by calendar year, sex, age, and neighbourhood deprivation quintile (*Q*) to generate the expected mortality, used in the relative survival analyses. Relative survival and excess mortality were estimated using the *strs* command in Stata (version 19; StataCorp, College Station, TX, USA).

Excess mortality and modelling

Excess mortality rates and excess mortality rate ratios (EMRRs) were derived from Poisson regression models fitted to observed and expected mortality. Analyses of one-year EMRRs included the entire cohort (i.e., from 2012–2022) and were adjusted for age group, sex, *Q*, aetiology, MALO status, and time period (i.e., 2012–2015, 2016–2019, and 2020–2022). Analyses of five-year EMRRs were restricted to individuals diagnosed between 2012 –2018 and adjusted for the same variables, except for the time period definition (i.e., 2012–2015 and 2016–2018), with the number of follow-up years included as an additional covariate.

Age-standardised incidence rates

Age-standardised incidence rates (ASIRs) of cirrhosis were calculated among individuals aged 40–74 years using direct standardisation to the 2013 Revised European Standard Population (ESP-13). The original ESP-2013 weights were truncated to exclude ages <40 and >74 years and rescaled proportionally to a total of 100,000 persons. Confidence intervals (CIs) for ASIRs were calculated as previously described. ASIRs were estimated separately by *Q*. Associations between socioeconomic deprivation and cirrhosis incidence were analysed using Poisson regression, with case data aggregated for each covariate and population counts entered as log-transformed offsets. Models were adjusted for sex, age group (40–44, 45–49, …, 70–74), and calendar year.

Excess deaths and absolute burden

To explore socioeconomic gradients in excess mortality, we estimated the number of excess deaths per 100,000 inhabitants during the five years following cirrhosis diagnosis, stratified by calendar year (i.e., 2012, 2015, and 2018), age group, and *Q*. These calendar years were chosen to represent the earliest (2012), midpoint (2015), and most recent (2018) cohorts with a complete five-year follow-up.

The number of excess deaths attributable to cirrhosis within each *Q* was calculated as:

$$Number of excess deaths in a Q = N_{Q}\times({ES}_{Q} -{OS}_{Q})$$

where *N_Q_* denotes the number of observed cirrhosis cases in a *Q*, *OS_Q_* the observed survival proportion in a *Q*, and *ES_Q_* denotes the expected survival proportion in the matched general population matched by age, sex, calendar year, and *Q*. This provides an estimate of the absolute number of deaths exceeding those expected from background mortality, quantifying the excess mortality attributable to cirrhosis within each socioeconomic stratum. These estimates were age-standardised to the ESP-13 and summarised visually as a heat-map, illustrating gradients in cirrhosis-related excess deaths across age, *Q*, and calendar year.

| Table S1. Sociodemographic characteristics | | |
| --- | --- | --- |
| Variable | **Definition** | **Source** |
| Sex | Male or Female (biological) | Statistics Sweden, NPR |
| Age | Age at cirrhosis diagnosis | Statistics Sweden, NPR |
| Region of birth | Nordic – Sweden, Denmark, Finland, Norway, Iceland, the Faroe Island, Greenland, and Åland | Statistics Sweden |
|  | Non-Nordic – all other countries |  |
| Educational level | Low – up to 9 years of formal education | Statistics Sweden |
|  | Medium – 10 to 12 years of formal education |  |
|  | High – more than 12 years of formal education |  |
| Household income | Based on the distribution of household incomes for all households^a^ in Sweden the year prior to cirrhosis diagnosis | Statistics Sweden |
|  | High – highest quartile, >75% (wealthiest) |  |
|  | Medium – second and third quartiles, 25-75% |  |
|  | Low – lowest quartile, <25% (poorest) |  |
| Neighbourhood deprivation level | Quintiles based on the proportion of individuals with a low household income per DeSO^b^ | Statistics Sweden |
|  | *Q1* – least deprived 🡪 *Q5* – most deprived |  |
| NPR=National Patient Register  ^a^ Household income refers to an individual’s disposable income per consumption unit. Disposable income includes all taxable and tax-exempt income, minus taxes and negative transfers, and accounts for capital gains or losses—for example, from the sale of stocks, mutual funds, or real estate. To ensure comparability across different household types, Statistics Sweden applies a consumption-weighting system based on household composition. This system adjusts disposable income by dividing it by a household’s consumption weight, which reflects expected economic needs. The scale, informed by budget calculations from the Swedish Consumer Agency, assigns weights as follows: 1.0 for a single adult, 1.51 for a cohabiting couple, 0.6 for each additional adult, 0.52 for the first child (aged 0–19), and 0.42 for each subsequent child.  ^b^ DeSO (Demografiska statistikområden) – or Demographic Statistical Areas – is a small-area geographic classification developed by Statistics Swedenin 2018 to improve local-level population and health statistics. DeSO divides Sweden into approximately 5,984 areas, each designed to include around 700–4,000 inhabitants (average about 1,600), depending on population density. The boundaries are constructed to be stable over time. | | |

| Table S2. Clinical characteristics | | |
| --- | --- | --- |
|  | **International Classification of Diseases, 10^th^ Edition (ICD-10) codes** | **Source** |
| Liver cirrhosis |  |  |
| Alcohol-related | K73.0 | NPR, CDR |
| Viral hepatitis-related | B18.1E/G, B18.2E/G | NPR, CDR |
| Unspecified | K74.6 | NPR, CDR |
| Oesophageal varices | I85.0, I85.9, I98.2, I98.3 | NPR |
| Gastric varices | I86.4 | NPR |
| Portal hypertension | K76.6 | NPR, CDR |
| Hepatorenal syndrome | K76.7 | NPR, CDR |
| Ascites | R18 and/or Procedural code TJA10 (only combined with ICD-10 code for liver disease) | NPR, CDR |
| Hepatic encephalopathy | ICD-10 code for liver disease + ATC-code A07AA11 (Rifaximin) | NPR, PDR |
| Aetiology |  |  |
| Viral hepatitis | B16, B17, B18, B19 | NPR, CDR |
| Alcohol-related liver disease | K70 or unspecified cirrhosis (K74.6) and ICD-10 codes associated with alcohol-use disorder: F10, E24.4, G62.1, I42.6, K29.2, G31.2, G72.1, K85.2, K86.0, T51.0, T51.9, Y57.3, X65, Z50.2, Z71.4, Z72.1 | NPR, CDR |
| Metabolic (excluding MASLD) | E83.1 (haemochromatosis), E83.0B (Wilson’s disease), E88.0A-B (alpha-1-antitrypsin deficiency) | NPR, CDR |
| Autoimmune liver disease | K75.4 (autoimmune hepatitis), K74.3 (primary biliary cholangitis), K83.0A (primary sclerosing cholangitis [PSC]). PSC was also defined in patients with inflammatory bowel disease (K50, K51, K52.3) and ICD-10 code for unspecified cholangitis (K83.0) | NPR |
| Metabolic dysfunction-associated steatotic liver disease (MASLD) | K76.0 (non-alcoholic fatty liver disease), K75.8 (non-alcoholic steatohepatitis) and no other code associated with viral hepatitis, ALD, other metabolic liver diseases, or autoimmune liver diseases. MASLD was also defined in patients with unspecified cirrhosis (K74.6) and coexisting diabetes or obesity | NPR |
| Unspecified | K74.6 and no other criteria described above fulfilled | NPR, CDR |
| MALOs^a^ |  |  |
| Ascites | R18 and/or Procedural code TJA10 | NPR, CDR |
| Variceal bleeding | I85.0, I98.2 | NPR, CDR |
| Hepatic encephalopathy | ATC-code A07AA11 (Rifaximin) | PDR |
| Hepatorenal syndrome | K76.7 | NPR, CDR |
| Liver transplantation | Z94.4 or Procedural codes JJC00, JCC10, JCC20, JCC30, JCC40, JCC96 | NPR, CDR |
| Hepatocellular carcinoma^b^ | C22.0 | NPR, NCR, CDR |
| Comorbidities |  |  |
| Arterial hypertension | I10-I15 | NPR |
| Diabetes | E10-E14; ATC: A10A (insulin), A10B (other glucose lowering drugs), A10X (other drugs against diabetes) | NPR, PDR |
| Obesity | E65, E66 | NPR |
| Hyperlipidaemia | E78; ATC: C10 (drugs affecting serum lipids) | NPR, PDR |
| Coronary artery disease | I20-I25; ATC: C01DA (organic nitrates) | NPR, PDR |
| Cerebrovascular disease | I60-I69 | NPR |
| Chronic kidney disease | N11, N18, I13.1, I13.2, I13.9, I12.0, Z94.0, T86.1, Z49, Z99.2 | NPR |
| Chronic obstructive pulmonary disease | J41-J44 (40 years and older) | NPR |
| Depression or anxiety | F30-F34, F38-F39, F41, F43 | NPR |
| Non-HCC cancer | C00-C97 (C22.0 excluded) diagnosed within 2 years prior to cirrhosis diagnosis |  |
| ATC: Anatomy Therapeutic Chemical; CDR: Cause of Death Register; MALOs: major adverse liver outcomes; NCR: National Cancer Register; NPR: National Patient Register; Prescribed Drug Register.  ^a^ MALOs registered within 90 days after a first ICD-10 code associated with cirrhosis were regarded as being present at baseline. ^b^ Hepatocellular carcinoma was regarded as present at baseline if diagnosed within 180 days. | | |

| Table S3. Baseline characteristics of 21,583 individuals aged 40–74 years diagnosed with cirrhosis in Sweden between 2012–2022, stratified by sex | | | | |
| --- | --- | --- | --- | --- |
|  | | **Sex** | |  |
|  | | **Male** | **Female** | **Total** |
|  | | 13966 (65) | 7617 (35) | 21583 (100) |
| Median age | | 63 (55–68) | 63 (56–69) | 63 (56–69) |
| Age group | |  |  |  |
| 40–44 | | 528 (4) | 246 (3) | 774 (4) |
| 45–49 | | 944 (7) | 447 (6) | 1391 (6) |
| 50–54 | | 1623 (11) | 803 (11) | 2426 (11) |
| 55–59 | | 2229 (16) | 1199 (16) | 3428 (16) |
| 60–64 | | 2789 (20) | 1473 (19) | 4262 (20) |
| 65–69 | | 3098 (22) | 1765 (23) | 4863 (22) |
| 70–74 | | 2755 (20) | 1684 (22) | 4439 (21) |
| Region of birth | | | | |
| Nordic | | 12151 (87) | 6635 (87) | 18786 (87) |
| Non-Nordic | | 1815 (13) | 982 (13) | 2797 (13) |
|  | Europe excl. Nordic | 805 (6) | 447 (6) | 1252 (6) |
|  | Asia | 611 (4) | 328 (4) | 939 (4) |
|  | Africa | 232 (2) | 107 (1) | 339 (2) |
|  | Other | 167 (1) | 100 (1) | 267 (1) |
| Educational level | |  |  |  |
| High (>12 years) | | 2628 (19) | 1626 (21) | 4254 (20) |
| Medium (10-12 years) | | 6936 (49) | 3843 (51) | 10779 (50) |
| Low (≤9 years) | | 4157 (30) | 1986 (26) | 6143 (28) |
| Unknown | | 245 (2) | 162 (2) | 407 (2) |
| Household income | |  |  |  |
| High | | 2684 (19) | 1380 (18) | 4064 (19) |
| Medium | | 6129 (44) | 3476 (46) | 9605 (45) |
| Low | | 5153 (37) | 2761 (36) | 7914 (37) |
| Neighbourhood deprivation | |  |  |  |
| Q1 (least) | | 1859 (13) | 1089 (14) | 2948 (13) |
| Q2 | | 2478 (18) | 1379 (18) | 3857 (18) |
| Q3 | | 2804 (20) | 1467 (19) | 4271 (20) |
| Q4 | | 3133 (22) | 1652 (22) | 4785 (22) |
| Q5 (most) | | 3692 (27) | 2020 (27) | 5722 (27) |
| Aetiology | | | | |
| Viral hepatitis | | 3779 (27) | 1535 (20) | 5314 (24) |
| ALD | | 6983 (50) | 2990 (39) | 9973 (46) |
| MASLD | | 1371 (10) | 1169 (15) | 2540 (12) |
| Autoimmune | | 429 (3) | 801 (11) | 1230 (6) |
| Metabolic^a^ | | 162 (1) | 64 (1) | 226 (1) |
| Unspecified | | 1242 (9) | 1058 (14) | 2300 (11) |
| Any MALO at diagnosis | | 6370 (46) | 3178 (42) | 9548 (44) |
| Hepatocellular carcinoma | | 1139 (8) | 273 (4) | 1412 (7) |
| Comorbidities | | | | |
| Arterial hypertension | | 5091 (36) | 2476 (33) | 7567 (35) |
| Type 2 diabetes | | 4461 (32) | 2140 (28) | 6601 (31) |
| Obesity | | 1555 (11) | 1104 (14) | 2659 (12) |
| Hyperlipidaemia | | 4504 (32) | 2287 (30) | 6791 (31) |
| Coronary artery disease | | 1681 (12) | 523 (7) | 2204 (10) |
| Cerebrovascular disease | | 826 (6) | 415 (5) | 1241 (6) |
| Chronic kidney disease | | 935 (7) | 381 (5) | 1316 (6) |
| COPD | | 1184 (8) | 836 (11) | 2020 (9) |
| Depression or anxiety | | 1153 (8) | 804 (11) | 1957 (9) |
| Non-HCC cancer | | 1150 (8) | 646 (8) | 1796 (8) |
| ALD=alcohol-related liver disease. COPD=chronic obstructive pulmonary disease. MALO=major adverse liver outcome. MASLD=metabolic dysfunction-associated steatotic liver disease.  ^a^ Including alpha-1-antitrypsin deficiency, haemochromatosis, and Wilson’s disease but not MASLD. | | | | |

| Table S4. Adjusted estimated excess mortality rate ratio (EMRR) at one and five years after cirrhosis diagnosis among individuals aged 40–74 years in Sweden, 2012–2022 | | | |
| --- | --- | --- | --- |
|  | **EMRR (95% confidence interval)** | | |
|  | **1 year** | | **5 years** |
| Age group |  | |  |
| 40–44 | 1.0 (reference) | | 1.0 (reference) |
| 45–49 | 1.22 (0.91–1.65) | | 1.19 (0.96–1.47) |
| 50–54 | 1.64 (1.25–2.16) | | 1.52 (1.25–1.84) |
| 55–59 | 1.78 (1.36–2.35) | | 1.70 (1.41–2.05) |
| 60–64 | 2.27 (1.73–2.98) | | 2.06 (1.71–2.48) |
| 65–69 | 2.74 (2.10–3.58) | | 2.41 (2.01–2.90) |
| 70–74 | 3.03 (2.32–3.96) | | 2.89 (2.40–3.48) |
| Sex | |  | |
| Female | 1.0 (reference) | | 1.0 (reference) |
| Male | 1.22 (1.12–1.33) | | 1.18 (1.11–1.26) |
| Neighbourhood deprivation |  | |  |
| Q1 (least) | 1.0 (reference) | | 1.0 (reference) |
| Q2 | 1.11 (0.96–1.30) | | 1.08 (0.97–1.20) |
| Q3 | 1.19 (1.03–1.38) | | 1.17 (1.05–1.30) |
| Q4 | 1.13 (1.02–1.30) | | 1.14 (1.03–1.26) |
| Q5 (most) | 1.21 (1.07–1.37) | | 1.18 (1.10–1.28) |
| Aetiology | |  | |
| Viral hepatitis | 1.0 (reference) | | 1.0 (reference) |
| ALD | 1.73 (1.54–1.94) | | 1.63 (1.50–1.76) |
| MASLD | 1.30 (1.11–1.51) | | 1.15 (1.03–1.28) |
| Autoimmune | 0.73 (0.59–0.90) | | 0.77 (0.66–0.89) |
| Metabolic^a^ | 1.21 (0.85–1.73) | | 1.16 (0.90–1.49) |
| Unspecified | 1.87 (1.59–2.20) | | 1.24 (1.10–1.39 |
| Period |  | |  |
| 2012–2015 | 1.0 (reference) | | 1.0 (reference) |
| 2016–2019^b^ | 0.89 (0.81–0.97) | | 0.93 (0.90–1.00) |
| 2020–2022 | 0.89 (0.79–0.99) | | Excluded |
| Follow- up year |  | |  |
| 1^st^ | Excluded | | 1.0 (reference) |
| 2^nd^ | Excluded | | 0.38 (0.35–0.41) |
| 3^rd^ | Excluded | | 0.34 (0.31–0.36) |
| 4^th^ | Excluded | | 0.32 (0.29–0.35) |
| 5^th^ | Excluded | | 0.30 (0.27–0.33) |
| EMRRs were estimated using relative survival models based on Poisson regression with robust standard errors. One-year analyses included individuals diagnosed between 2012–2022, while five-year analyses were restricted to those diagnosed between 2012–2018. Models were adjusted for all variables presented in the table. Expected mortality was derived from population life tables matched by age, sex, calendar year, and neighbourhood deprivation quintile.  ALD=alcohol-related liver disease. MASLD=metabolic dysfunction-associated steatotic liver disease.  ^a^ Including alpha-1-antitrypsin deficiency, haemochromatosis, and Wilson’s disease but not MASLD.  ^b^ Calendar period 2016–2018 in the five-year EMRR model. | | | |
